# A cancer-derived mutation in the PSTAIRE helix of cyclin-dependent kinase 2 alters the stability of cyclin binding

**DOI:** 10.1016/j.bbamcr.2010.04.004

**Published:** 2010-07

**Authors:** Emma S. Child, Tereza Hendrychová, Karen McCague, Andy Futreal, Michal Otyepka, David J. Mann

**Affiliations:** a Department of Life Sciences, Imperial College, South Kensington, London SW7 2AZ, UK; b Department of Physical Chemistry, Faculty of Science, Palacky University, Olomouc, Czech Republic; c Chemical Biology Centre and Department of Chemistry, Imperial College, South Kensington, London SW7 2AZ, UK; d Cancer Genome Project, Wellcome Trust Sanger Institute, Hinxton, CB10 1SA, UK

**Keywords:** Cyclin-dependent kinase, cdk, PSTAIRE, p21Cip1, p27Kip1, cdc28

## Abstract

Cyclin-dependent kinase 2 (cdk2) is a central regulator of the mammalian cell cycle. Here we describe the properties of a mutant form of cdk2 identified during large-scale sequencing of protein kinases from cancerous tissue. The mutation substituted a leucine for a proline in the PSTAIRE helix, the central motif in the interaction of the cdk with its regulatory cyclin subunit. We demonstrate that whilst the mutant cdk2 is considerably impaired in stable cyclin association, it is still able to generate an active kinase that can functionally complement defective cdks in vivo. Molecular dynamic simulations and biophysical measurements indicate that the observed biochemical properties likely stem from increased flexibility within the cyclin-binding helix.

## 1 Introduction

The central regulators of mammalian cell proliferation are the cyclin-dependent kinases (cdks) that phosphorylate key substrates in a sequential manner to facilitate orderly progression through the cell cycle [1,2]. Commitment to division is determined in the G1 phase of the cell cycle, a time when the predominantly active cdks are cdk2 and cdk4 [2]. These cdks are activated sequentially in part through interaction with their regulatory subunits, the D-type cyclins binding to cdk4 early in G1 phase, with E-type cyclins associating with cdk2 in mid-G1, and with A-type cyclins combining with cdk2 in S phase [2]. Genetic studies have largely validated this view although they have also uncovered considerable functional redundancy amongst the cdks [1].

Association of cyclins with their cognate cdk subunit typically involves interaction over a large surface area that is centred on the so-called PSTAIRE helix of the cdk [3,4], this being equivalent to the C helix in protein kinase A [5]. In associating with cdk2, E-type cyclins cause an essential rearrangement of the PSTAIRE helix to a conformation favouring the full activation of the cdk (the so-called ‘helix in’ arrangement) [6]. Together with the phosphorylation of a key threonine in the activation segment (also known as the T loop) adjacent to the catalytic site, the PSTAIRE helix rearrangements upon cyclin binding lead to the realignment of essential ATP binding residues into an active conformation.

Because of their central regulatory role in proliferative control, cdks (along with other signal transducing protein kinases) have become the focus of much interest in the study of diseases affecting proliferation, such as cancer. In order to gain insight into mutations that may affect kinase function, large-scale sequencing projects have been directed at identifying abnormalities in the fraction of the genome encoding the protein kinases in cancerous tissue [7]. Such studies have the power to uncover many mutations in focussed screens of interesting candidate genes although they do not in themselves address the functional consequences of detected mutations. Here we describe the characterisation of the properties of one missense mutation in cdk2 detected by the Cancer Genome Project that alters the proline of the PSTAIRE helix to a leucine in a glioblastoma cell line.

## 2 Materials and methods

### 2.1 Antibodies

The following antibodies were used: cyclin E1 (HE12), p21 (C-19), p27 (C-19) and cdk2 (sc-163) from Santa Cruz and anti-Flag (M2) antibody from Sigma. Anti-cyclin A2 (E65.1) was from Cancer Research UK. HRP-conjugated secondary antibodies (mouse, rabbit and goat) were from Jackson ImmunoResearch.

### 2.2 Plasmids and viruses

The P45L mutant cdk2 was generated by site-directed mutagenesis and, after sequence verification, subcloned into pBlueBacHis A (Invitrogen). This was transfected into Sf9 cells with linearised viral DNA (BaculoGold, BD Biosciences) and recombinant virus amplified to generate high titre baculovirus stock. The P45L mutant cdk2 was also cloned into pcDNA3 for mammalian expression. In addition, the P45L mutant cdk2 was cloned into the yeast two-hybrid vector pGBDU-C1 [8]. Control constructs/viruses were created with wild type cdk2. Other recombinant baculoviruses encoding full-length cyclins E1 and A2 have been described [9]. Coding sequences for p21 and p27 were cloned into pRSET and expressed in bacteria. Proteins were purified by metal immunoaffinity purification following treatment of sonicated lysates at 65 °C for 10 min and removal of the precipitated material.

### 2.3 Cell culture

Sf9 cells were grown in Graces insect media (PAA) supplemented with 10% foetal bovine serum (PAA) and maintained at 27 °C. The Sf9 cells were infected with baculoviruses directing the expression of wild type or P45L mutant cdk2, cyclin A2, cyclin E1 and/or wild type virus at multiplicities of infection greater than 10:1. U2OS cells were grown in DMEM supplemented with 10% foetal bovine serum in a humidified atmosphere containing 10% carbon dioxide in air at 37 °C. The cells were transfected with pcDNA3-cdk2 and cdk2 P45L by calcium phosphate precipitation [9]. The transfected cells were washed 24 h later and after a further 24 h the cells were harvested.

### 2.4 Yeast methods

*Saccharomyces cerevisiae* strain Y246a (*MATa cdc28-4 trp1 ura3-52 tyr1*; a gift from John Diffley) was transformed with plasmids using lithium acetate. Transformants were plated on selective media and incubated at permissive (30 °C) or restrictive (35 °C) temperatures for three days.

### 2.5 Kinase assays

Sf9 cells infected with the appropriate recombinant baculoviruses were lysed 72 h post infection by hypotonic lysis using kinase buffer (25 mM Hepes pH7.9/5 mM MgCl/0.1% 2-mercaptoethanol/0.1 mM EDTA) for 10 min on ice. Cell debris was removed by centrifugation. The cell lysate was used in kinase assays as described below. In immunoprecipitation kinase assays, immunoprecipitations were performed at 4 °C for 2 h using protein A-Sepharose beads, cdk2 antibody (0.4μg) in PBS/0.1% Tween 20. The beads were washed three times in PBS/0.1% Tween 20, followed by two further washes in kinase buffer. Kinase assays were performed in kinase buffer containing 0.5–1 μg Histone H1 or GST-pRb, 100 μM ATP and 2.5 μCi [γ^32^P]-ATP and incubated for 30 min at 30 °C. Reactions were terminated by the addition of an equal volume of 2× SDS-PAGE loading buffer and resolved by SDS-PAGE and then subject to autoradiography.

### 2.6 Immunoprecipitations

Cells were washed in PBS and then lysed in 0.1% Tween 20/150 mM NaCl/1 mM DTT/50 mM Tris–HCl pH8.0/1 mM EDTA/2.5 mM EGTA/50 mM NaF at 4 °C by passing the lysate five times through a 21 gauge needle. Debris was removed by centrifugation. Lysates were then immunoprecipitated as described above. The precipitates were washed three times in lysis buffer, resuspended in 2× SDS-PAGE loading buffer and immunoblotted with detection via chemiluminescence visualised on Fuji Image Reader LAS-3000.

### 2.7 Circular dichroism

A Chirascan spectropolarimeter (Applied Photphysics) was used to measure the CD spectra and temperature-dependent protein unfolding profiles [10]. A 0.1 cm path length was used with a protein concentration of 0.1 mg/ml in buffer containing 10 mM Tris–HCl pH 8.0/150 mM NaCl/10% glycerol/1 mM DTT. *T*_m_ was determined at 230 nm with temperature range from 20 to 80 °C (recorded every 0.5 °C).

### 2.8 Thermal shift assays

Sypro Orange™ (Invitrogen) was added to a solution of cdk2 (5–10 μg) in 20 or 40 mM Tris–HCl pH8.0/150 mM NaCl/1 mM DTT/5% glycerol (total volume 95 μl) in a 96-well plate. The samples were heated from 20 to 90 °C at intervals of 0.5 °C, each held for 10 s on an IQ5 Real-Time PCR Detection System (BioRad). The fluorescence intensity was measured at excitation/emission wavelengths of 485/530 nm and the *T*_m_ was calculated as the temperature of maximum inflection of the melting curve.

### 2.9 Computational methods

Molecular dynamics simulations were carried out using the program PMEMD (from AMBER 9.0 package) [11] with the *parm99* and *ff03* force fields [12,13]. The *ff03* force field is the most recent version of AMBER force field with revised *ψ*/*φ* torsion parameters and recalculated atomic partial charges from the electrostatic potential in the continuum (*ε*_r_ = 4) produced by the B3LYP/cc-pVTZ method. The *parm99* force field does not accurately represent glycine behavior and that is prone to other inaccuracies including over-stabilisation of α-helical peptide conformations and over-estimation of β-bend propensity. Nonetheless, both force fields are believed to perform well on compact and folded protein structures. The cdk2 starting structure (PDB code: 2CLX, resolution 1.8 Å with the ligand removed from the active site [14]) and the starting structure for pT160-cdk2/ATP complexed with Cyclin E was taken from PDB (PDB code: 1W98, resolution 2.15 Å [6], where ATP and Mg^2+^ was added from 1JST structure [15]). The applied MD simulation protocol, which has been repeatedly successfully used to study cdk2 systems [16–19], was as follows. First, the protonation states of all histidines were checked using WHATIF [20] to create an optimal H-bond network. All hydrogens were added using the Xleap program from the AMBER 9.0 package. The structures were neutralized by adding 4 Cl^−^ counter ions to the monomeric cdk2 systems and 7 Cl^−^ counter ions to the systems complexed with cyclin E. Each system was solvated in a rectangular water box with a layer of water molecules 9 Å thick. The energy of each system was then minimized as follows prior to the main molecular dynamics simulation run. The protein was frozen and the solvent molecules and counterions were allowed to move during a 1000 step minimization and a 10 ps long molecular dynamics run under NpT conditions. The side chains were then relaxed using several sequential minimizations with the force constants applied to the backbone atoms being decreased in each run. Following this relaxation, the system was 20 ps heated from 10 to 50 K, then 70 ps from 50 to 298.16 K and the thermalization was ended by 10 ps warming at 298.15 K. The production phases were run for 10 ns for all systems. Free cdk2 system studied comprised ∼ 35 000 atoms and cdk2-cyclin E systems comprised ∼ 70 000 atoms. The simulation period was chosen as a compromise between the quality of conformation space sampling and the calculation length. Time integration steps of 2 fs were used, together with particle-mesh Ewald (PME) methods for electrostatic interactions. All simulations were run under periodic boundary conditions in the NpT ensemble at 298.16 K and at a constant pressure of 1 atm. The SHAKE algorithm with a tolerance of 10^− 5^ Å was applied to fix all bonds containing hydrogen atoms. Non-bonding interactions were subject to a 9.0 Ǻ cut-off. Coordinates were stored every 2 ps. All analyses of the MD simulations were carried out using the PTRAJ modules of AMBER 9.0.

## 3 Results

During large-scale sequencing to identify mutations in protein kinases in human cancer cells and primary tissue, we identified a heterozygous missense mutation in the cdk2 gene in a short-term culture of a glioblastoma (data not shown). The mutation was in the cdk2 kinase at position 45, altering the conserved proline of the cyclin-binding PSTAIRE motif to a leucine (P45L, hereafter). This motif is characteristic of many cdks and is central to the binding of the activating cyclin subunit to the kinase [4]. Inspection of the crystal structures of cdk2/cyclin E1 [6] revealed that the proline side chain was oriented away from the cyclin subunit (not shown) making direct side chain effects on cyclin binding unlikely. However, given the importance of this region in regulating cdk activity, the presence of this mutation in a cancer cell line, the constraints imposed by proline in terms of peptide backbone angles and the non-conservative nature of the substitution involved, we undertook to characterise the properties of this P45L cdk2 to determine if they could be contributory to deregulated cell cycle control.

**Fig. 1:**
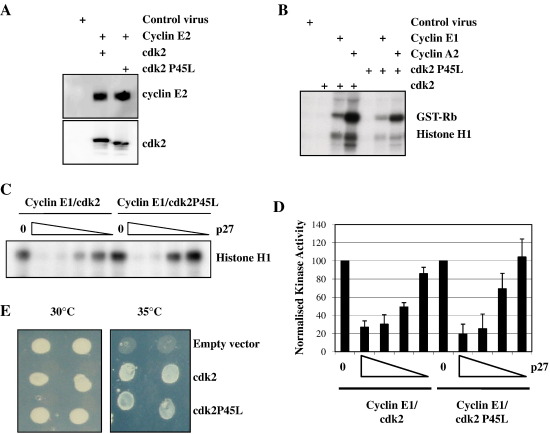
P45L cdk2 is functional as a protein kinase. A Sf9 cells were co-infected with recombinant baculoviruses encoding wild type cdk2 or P45L cdk2 plus cyclin E1 or were infected with non-recombinant baculovirus. Cell lysates were immunoblotted as indicated. B Sf9 cells were infected with the indicated recombinant baculoviruses, lysed after 72 h and assayed for kinase activity against both GST-Rb and Histone H1 using [γ-^32^P]ATP followed by autoradiography. C and D Lysates as described in A were incubated with increasing concentrations of p27 purified from recombinant bacteria for 10 min at room temperature and then incubated for 30 min at 30 °C in the presence of Histone H1 and [γ-^32^P]ATP. 0 denotes assays performed in the absence of p27. Assays were performed in triplicate and quantified by Phosphorimage analysis. The bar chart shows the means and standard deviation with activities normalised to the uninhibited control. E *S. cerevisiae* with a temperature sensitive allele of *cdc28* were transformed with plasmids expressing either wild type or P45L cdk2 or the parental vector and tested for their ability to proliferate at 30 °C and the restrictive 35 °C. Two individual transformants are shown for each plasmid.

Initially, we constructed a recombinant baculovirus directing expression of the mutant form of cdk2. This virus produced somewhat lower levels of recombinant protein than the wild type cdk2-encoding virus and the protein displayed a slightly enhanced mobility on SDS-PAGE (Fig. 1A) perhaps indicative of differences in retained secondary structure during electrophoresis. Co-infection of Sf9 cells with either recombinant baculovirus directing expression of wild type or P45L cdk2 and either an A- or E-type cyclin was performed and the kinase activity of the cell lysates assessed. Fig. 1B demonstrates that both forms of cdk2 generated kinase activity towards both Histone H1 and the Retinoblastoma protein, Rb. The mutant enzyme produced slightly less kinase activity than the wild type but this parallelled its reduced expression. The activities of both wild type and P45L cdk2 in complex with cyclin E1 (or cyclin A2) were equally sensitive to the cdk inhibitors p21 and p27 when titrated into the in vitro assays (Fig. 1C and D and Fig. S1).

To further demonstrate the functionality of the P45L mutant cdk2, we expressed it in *S. cerevisiae* containing a temperature sensitive allele of *cdc28* and tested for complementation at the restrictive temperature. As shown in Fig. 1E, P45L cdk2 enabled proliferation at the non-permissive temperature although proliferation was generally less robust than that of yeast expressing wild type cdk2 in agreement with previous observations [21]. Taken together, these experiments demonstrate that the P45L mutation in cdk2 is seemingly well tolerated and the kinase produced had gross properties that were similar to its wild type counterpart being functional both in vitro and in vivo.

**Fig. 2:**
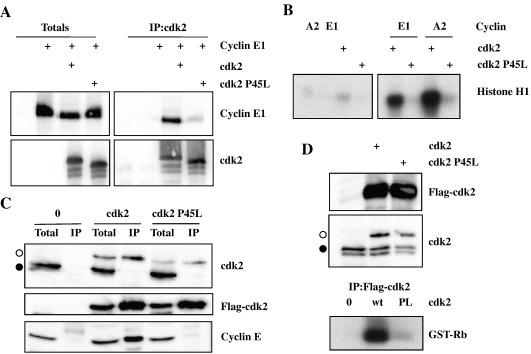
P45L cdk2 fails to stably associate with cyclins. A and B Sf9 cells were infected with the indicated recombinant baculoviruses, lysed after 72 h and immunoprecipitated through cdk2. Total cell lysates and immunoprecipitates were immunoblotted as indicated (A) or immunoprecipitates were used in kinase assays with Histone H1 as substrate (B). C and D U2OS cells were transfected with Flag-tagged wild type or P45L cdk2 and cell lysates were harvested and immunoprecipitations were performed through the Flag tag of the cdk2. Total lysates (Total) and immunoprecipitates (IP) were immunoblotted for the Flag epitope and total cdk2 showing exogenous (white circle) and endogenous (black circle) expression and cyclin E. Kinase assays against GST-Rb were also performed (D, lower panel).

Whilst the data suggested that the P45L cdk2 was functional, this mutant version of the enzyme appeared somewhat less efficient that the wild type equivalent in terms of expression in Sf9 cells, kinase activity and rescue of the phenotype in cdc28^ts^ yeast. In order to understand these observations further, we investigated the interaction of cyclins with this mutant cdk. Initially we used the baculovirus system to generate cell lysates expressing both cdk2 (wild type or P45L mutant) and cyclin E1. Lysates were immunoprecipitated through cdk2 and immunoblotted for the associated cyclin. As shown in Fig. 2A, cyclin E1 was readily detected in association with wild type cdk2 but only present at very low levels in the P45L cdk2 immunoprecipitates despite being present in the initial lysate at levels similar to the wild type protein. Further, immunoprecipitation kinase assays also demonstrated a lack of activity associated with the mutant kinase (Fig. 2B) when compared to its wild type counterpart.

To validate this data, we performed similar experiments in human cells. U2OS cells were transfected with plasmids directing expression of Flag-tagged cdk2 (wild type or P45L mutant) and immunoprecipitated through the epitope tag. Immunoblotting the precipitates again demonstrated that the P45L mutant cdk2 failed to stably associate with cyclin E whereas this cyclin was readily detectable in the precipitates with the wild type cdk2 construct (Fig. 2C). Similar results were obtained when the immunoprecipitation was performed through either cyclin E1 or A2 (Fig. S2). This difference was also observed in immunoprecipitation kinase assays (Fig. 2D).

**Fig. 3:**
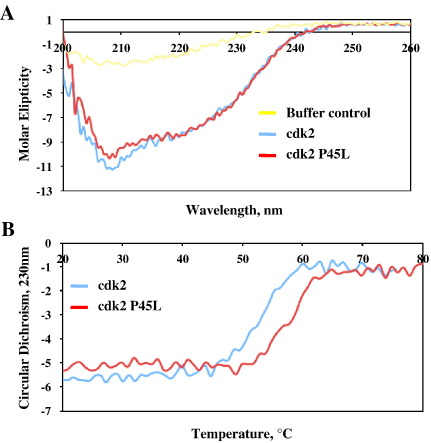
P45L cdk2 is more stable than its wild type counterpart. A CD spectra of cdk2 (blue) and P45L cdk2 (red) at 20 °C from 200 to 260 nm. The yellow line delineates the buffer control. B Denaturation curves for cdk2 (blue) and P45L cdk2 (red) at 230 nm from 20 to 80 °C. For all CD data, representative plots are shown from three independent repeats.

This biochemical evidence suggested that the P45L cdk2 was impaired in stable interaction with the cyclin subunit. We hypothesized that the mutation of the proline in the PSTAIRE helix may increase the flexibility in that region of the protein allowing the cdk to sample a greater ensemble of structures not all of which were permissive for cyclin binding. We reasoned that such an effect may be observed in a change in the thermal stability of cdk2. To test this idea we isolated poly(His)-tagged cdk2 (wild type and P45L mutant) from recombinant bacteria and performed circular dichroism (CD) and thermal denaturation assays. CD revealed wild type and P45L cdk2 proteins had very similar spectra indicating no gross differences in protein secondary structure (Fig. 3A). We next assessed cdk2 thermal stability by CD at 230 nm between 20 °C and 80 °C. From three separate determinations, wild type cdk2 melted at a lower temperature than the P45L mutant cdk2 (*T*_m_ 53.7 °C vs 58.1 °C, mean of two separate determinations; Fig. 3B). We verified this difference in a second thermal stability test, this time with a fluorescence-based assay using the environmentally sensitive dye, Sypro Orange. Again, the mutant protein was significantly more thermostable than the wild type (*T*_m_ 51.15 ± 0.13 °C vs 52.03 ± 0.35 °C, mean ± SD, *P* = 0.0151 by *t* test; Fig. S3).

To further elucidate the role of the P45L mutation on cdk2 and cdk2/cyclin E complex structure and dynamics, we performed molecular dynamics (MD) simulations of the following systems (see Table 1 for overview): monomeric wild type or P45L mutant cdk2 and both kinases (the threonine 160 phosphorylated form) in complex with cyclin E1 (and ATP). The convergence and the stability of the MD trajectories were documented by time evolution of root-mean-square deviation (RMSD) of heavy atoms from the X-ray structures (Fig. S4). The RMSD of all systems become balanced after ∼ 3 ns then systems reached their equilibrated states and remained fluctuating around the average values (Table 1). These RMSDs show that MD simulations were convergent and ready for further structural analyses.

**Fig. 4:**
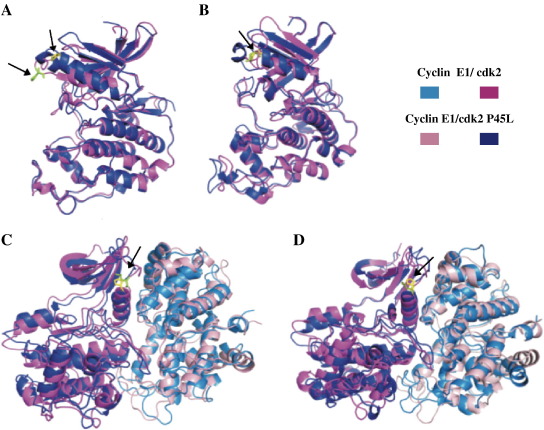
Molecular dynamics simulations of cdk2 variants. Images showing the average structures over the last 1 ns of simulations with proteins coloured according to the key. Amino acid 45 of the cdk2 is indicated by arrows in each panel, with the proline coloured yellow and the leucine green. A *parm99* and B *ff03* depict monomeric wild type and P45L cdk2. C *parm99* and D *ff03* depict cyclin E1/cdk2 complexes.

**Fig. 5:**
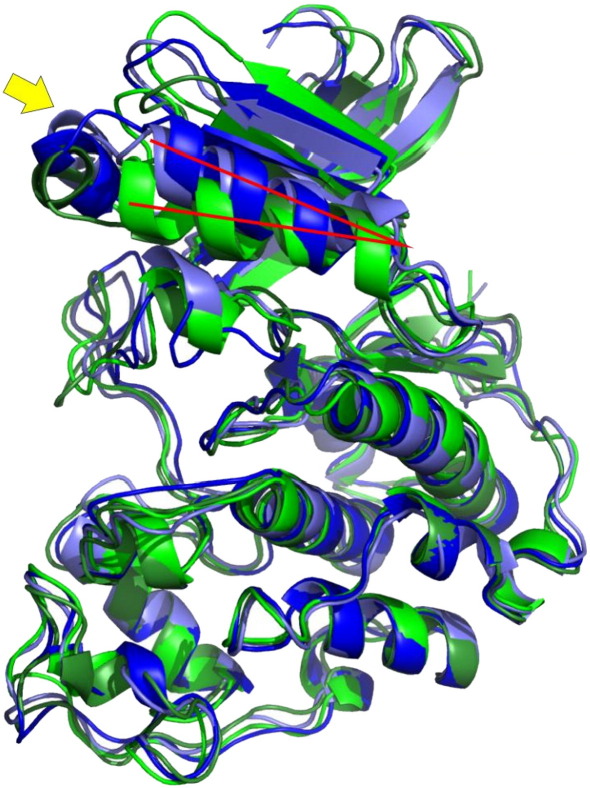
Structural differences predicted between wild type and P45L cdk2. Superposition of monomeric cdk2 structures averaged over the last ns of MD simulations, cdk2 (*parm99*) in dark blue, cdk2 (*ff03*) in light blue, P45L cdk2 (*parm99*) in dark green and P45L cdk2 (*ff03*) light green. The yellow arrow indicates the β3-PSTAIRE loop and an angle between the axes of PSTAIRE and LSTAIRE helices (red lines) is 16°.

The typical bilobal kinase fold is retained in all structures (Fig. 4). The largest differences between wild type cdk2 and P45L cdk2 occurred in the PSTAIRE helix, which contacts the regulatory cyclins and β3-PSTAIRE helix connecting loop. (A number of exposed loops also showed substantial structural deviations but given the inherent flexibility of such regions [18] we did not consider them further.) Residue P45 at the N-terminus of PSTAIRE helix in wild type cdk2 is partially exposed to the solvent. In the crystal structure of the cdk2/cyclin E1, the PSTAIRE helix contacts cyclin E1 and adopts its rotated inner conformation, which allows hydrogen bond between E51 from PSTAIRE and K31 and thus the P45 residue is rotated into the cdk2 protein too. The mutant structures of monomeric cdk2 differed in both PSTAIRE helix position and β3-PSTAIRE loop conformation from wild type structures (Fig. 5). The PSTAIRE helix of the mutant structures is rotated from its native position by 16 degrees (Fig. 5). While β3-PSTAIRE loop conformations of the wild type align well, the loop conformations of the P45L mutant differed from the wild type significantly, one being localized below and one above the conformation of the wild type loop, indicating greater flexibility of this region in agreement with analysis of temperature *B*-factors.

Temperature *B*-factors (*B*_f_) provide information about thermal fluctuations of amino acids. The *B*_f_ values were calculated from the last 1 ns of the MD simulations (Fig. S5). In all cases, *B*_f_ values of the PSTAIRE helix (residues 45–59) were higher for P45L mutants than for the wild types in agreement with the increased flexibility of the P45L cdk2 in this region. The *B*_f_ values of both wild type cdk2/cyclin E1 and P45L cdk2/cyclin E1 systems were similar to one another differing in flexibility within the activation segment (*parm99* simulation, maximum at residue 156) of cdk2 (Table 1). More variation was observed in the flexibility of the cyclin E1 associated with wild type cdk2: residues 252–256 which are oriented toward the activation segment of cdk2 and residues 339–347, a solvent exposed flexible loop lying adjacent to residues 252–256 (Fig. S5). The *B*_f_ of P45 and L45 does not substantially differ in cyclin-bound cdk2 with both being lower than *B*_f_s of the respective residues of monomeric cdk2.

**Fig. 6:**
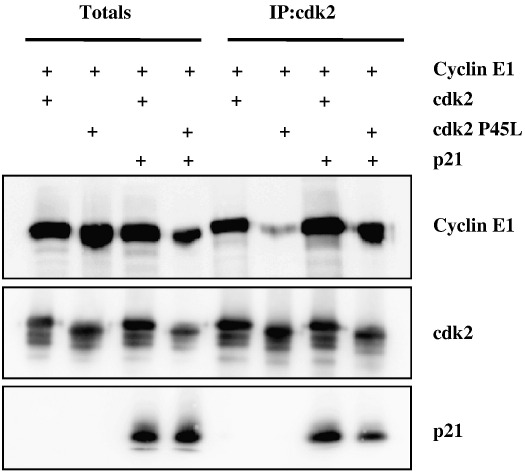
CIP/KIP inhibitors can stabilise the P45L cdk/cyclin E1 complex. Sf9 cells were co-infected with recombinant baculovirus encoding cyclin E1, wild type cdk2 or P45L cdk2 in the absence and presence of p21-encoding virus. Cell lysates were immunoprecipitated through cdk2 and immunoprecipitations were immunoblotted as indicated.

The rotated position of PSTAIRE helix and increased flexibility around the PSTAIRE helix in P45L cdk2 suggested by the MD simulations could provide an explanation for the reduced binding of this cdk with cyclins observed biochemically. The cdk inhibitors p21 and p27 have been shown to help in the assembly and stabilisation of D-type cyclin/cdk complexes [22,23]. Given the lack of stable interaction between the P45L cdk2 and cyclins, we asked if p21 could stabilise this cdk/cyclin interaction. Again, Sf9 cells were infected with baculovirus directing expression of cdk2 (wild type or mutant) and cyclin E1 in the absence or presence of p21-encoding virus. Cells were lysed and immunoprecipitated through the cdk and immunoblotted for the cyclin and p21. Fig. 6 clearly demonstrates that p21 was able to substantially stabilise the mutant cdk/cyclin interaction. Similar results were obtained with cyclin A2 (not shown).

## 4 Discussion

Activating mutations in protein kinases are increasingly recognised as driver mutations in various forms of cancer [7]. We have analysed the P45L mutation in the cell cycle regulator cdk2 found through targeted genomic sequencing of a glioblastoma and affecting the PSTAIRE cyclin-binding helix characteristic of cdks. The mutation does not appear to cause gross alterations to the cdk2 structure as indicated by the retention of biological activity and the similarity of the wild type and mutant CD spectra. Our analysis reveals interesting features of cdk2 biochemistry but does not suggest that this mutation is likely to be causal for cancer.

The interaction of cyclins and cdks relies upon a large interface (3252 Å^2^ for cyclin E1/cdk2 [6]) centred on the PSTAIRE helix of the cdk subunit. In the crystal structure of cyclin E1/cdk2 [6] and cyclin A2/cdk2 [4,15], the PSTAIRE helix lies almost parallel to the α5 helix of the cyclin and this cyclin helix is also adjacent to the loop connecting β3 and the PSTAIRE helix. These closely aligned regions make numerous hydrophobic interactions and hydrogen bonds in the cyclin E1/cdk2 and cyclin A2/cdk2 crystal structures [4,6]. The major difference observed biochemically between the wild type and P45L cdk2 proteins was the reduced ability of the mutant to maintain association with its cognate cyclin over prolonged periods. MD simulations indicate that the P45L mutation leads to a greater degree of flexibility in the β3-PSTAIRE helix loop as well as a displacement of the PSTAIRE helix in the monomeric kinase subunit and these analyses provide a potential explanation for the reduced stable association of this mutant kinase with its regulatory cyclin subunits observed experimentally.

Despite the lack of stable cyclin binding, P45L cdk2 displayed enzymatic activity very similar to the wild type kinase when assayed in lysates of recombinant baculovirus-infected Sf9 cells (the P45L mutant cdk2 retained ∼ 62% of the activity of wild type cdk2 in an Sf9 cell lysate although this activity is almost entirely lost during immunoprecipitation). One explanation for this observation could be a weaker, transient association between cyclin and cdk favoured in lysates over-expressing cyclins and cdks allowing occasional activation. Alternatively, it may be possible that the kinase assay conditions (high levels of ATP, protein substrate and kinase components) may stabilise the active cyclin/cdk complex permitting substrate phosphorylation whereas the immunoprecipitation assay conditions (no added ATP, dilute components) are not favourable for complex stability. The in vivo rescue of yeast expressing a temperature sensitive variant of CDC28 also provides compelling evidence that P45L cdk2 is functional in a cellular setting, delivering sufficient catalytic activity to allow cell cycle progression.

The ability of the cdk inhibitor p21 to facilitate stable complex assembly between the P45L cdk2 and a cyclin is reminiscent of the dual role of p21 in the activation of D-type cyclin/cdks [22,23]. However, to our knowledge, there are no reports of cdk2 kinase activity being sustained in the face of cdk inhibitor binding binding making this is an unlikely mechanism to account for the kinase activity we observed in cell lysates or the rescue of proliferation in the cdc28^ts^ yeast. However, it is possible that other cellular chaperones account for the assembly activity required for these events; it is known, for example, that D-type cyclin/cdk complexes have assembly factors other than p21 and p27 [24,25] and such mechanisms may account for P45L cdk2/cyclin complexation, with chaperones perhaps facilitating short-term interactions that are not maintained during the extended periods of immunoprecipitation experiments.

Isolation of the P45L mutant of cdk2 from a glioblastoma raised the possibility that the mutation could be a contributory factor to the aetiology of the disease. Our findings on the properties of the protein suggest that this is most likely not to be the case. Although the mutation produces a kinase that is functional in vitro and in vivo, the enzyme is impaired in its ability to form a stable association with its activating cyclin subunits. Thus, this mutation alone is unlikely to be a driver of oncogenesis, although in the context of the genomic instability associated with cancer, it is possible that other genetic alterations may influence its function.

## Figures and Tables

**Table 1:** Summary of molecular dynamics simulations.

System	Force field	Duration	Mean RMSD	B_f_ PSTAIRE Helix (aa 45–59)	B_f_ T Loop(aa 157–163)
		(ns)	(Å)	(Å^2^)	(Å^2^)
Wild type cdk2	*parm99*	10	1.9 ± 0.1	14.4	17.4
	*ff03*	30	1.7 ± 0.1	14.3	16.7
P45L-cdk2	*parm99*	30	1.9 ± 0.1	16.4	30.1
	*ff03*	30	1.7 ± 0.1	23.7	33.3
pT160-cdk2/ATP/cyclin E1	*parm99*	10	3.7 ± 0.2	6.1	11.9
	*ff03*	30	3.2 ± 0.2	4.2	6.2
pT160-P45L-cdk2/ATP/cyclin E1	*parm99*	10	1.8 ± 0.2	6.8	5.1
	*ff03*	30	3.2 ± 0.1	6.0	5.5

## Appendix A Supplementary data

Figure S1 A and B. Sf9 cells were co-infected with recombinant baculovirus encoding the indicated components and cells were lysed 72 h later. Increasing concentrations of p21 or p27 were added to the cell lysates, the mixtures incubated for 30 min at 30 °C followed by kinase assay with GST-Rb or Histone H1 as substrate. Cont. indicates Sf9 cells were infected with wild type baculovirus only. C. Representative immunoblots for cyclin A2 and cdk2 variants used in the assays. D. Immunoblots showing the presence of p21 and p27 added to the inhibition assays; 0 denotes control purification from non-expressing bacteria.

Figure S2 U2OS cells were transfected with Flag-tagged wild type or P45L cdk2 or empty vector. Cell lysates were harvested and immunoprecipitations were performed through either cyclin A2 or cyclin E1. Totals and immunoprecipitations (IP) were immunoblotted for the Flag epitope (cdk2).

Figure S3 Thermal denaturation assays were performed with recombinant His-tagged cdk2 (A wild type, B P45L) using the environmentally sensitive dye Sypro Orange. Graphs show data in triplicate.

Figure S4 The root-mean-square deviation (RMSD) from 0 to 30 ns of CDK2 systems at *parm99* (A) and CDK2 systems with cyclin E at *parm99* (B), CDK2 systems at *ff03* (C) and CDK2 with cyclin E at *ff03* (D). Wild type cdk2 is in blue with P45L cdk2 in magenta.

Figure S5 The temperature *B*-factors (*B*_f_) of CDK2 systems at *parm99* (A) and CDK2 systems with cyclin E at *parm99* (B), CDK2 systems at *ff03* (C) and CDK2 with cyclin E at *ff03* (D). Wild type cdk2 is in blue with P45L cdk2 in magenta.
