# Supplementary material for: A cancer-derived mutation in the PSTAIRE helix of cyclin-dependent kinase 2 alters the stability of cyclin binding
Source: Biochim Biophys Acta. 2010 Jul;1803(7):858–64. doi: 10.1016/j.bbamcr.2010.04.004 (PMC3111755; doi:10.1016/j.bbamcr.2010.04.004)
Supplement: Figure S1 — A and B. Sf9 cells were co-infected with recombinant baculovirus encoding the indicated components and cells were lysed 72 h later. Increasing concentrations of p21 or p27 were added to the cell lysates, the mixtures incubated for 30 min at 30 °C followed by kinase assay with GST-Rb or Histone H1 as substrate. Cont. indicates Sf9 cells were infected with wild type baculovirus only. C. Representative immunoblots for cyclin A2 and cdk2 variants used in the assays. D. Immunoblots showing the presence of p21 and p27 added to the inhibition assays; 0 denotes control purification from non-expressing bacteria. [file mmc1.ppt]

## Slide 1
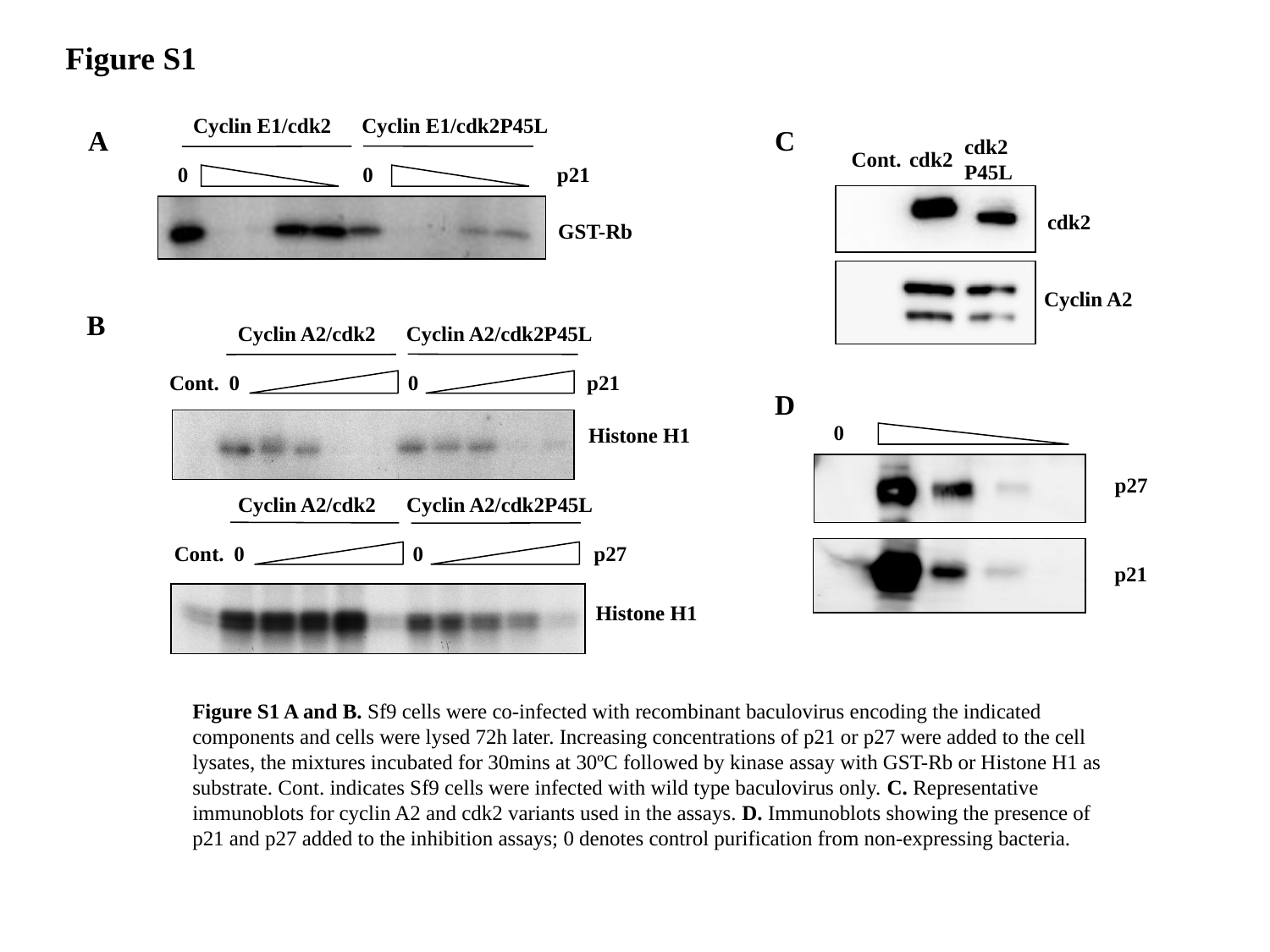

Figure S1
Cyclin E1/cdk2
Cyclin E1/cdk2P45L
A
C
cdk2
P45L
Cont.
cdk2
cdk2
Cyclin A2
0
0
p21
GST-Rb
B
Cyclin A2/cdk2
Cyclin A2/cdk2P45L
Cont.
0
0
p21
D
0
Histone H1
p27
Cyclin A2/cdk2
Cyclin A2/cdk2P45L
Cont.
0
0
p27
p21
Histone H1
Figure S1 A and B. Sf9 cells were co-infected with recombinant baculovirus encoding the indicated components and cells were lysed 72h later. Increasing concentrations of p21 or p27 were added to the cell lysates, the mixtures incubated for 30mins at 30ºC followed by kinase assay with GST-Rb or Histone H1 as substrate. Cont. indicates Sf9 cells were infected with wild type baculovirus only. C. Representative immunoblots for cyclin A2 and cdk2 variants used in the assays. D. Immunoblots showing the presence of p21 and p27 added to the inhibition assays; 0 denotes control purification from non-expressing bacteria.

## Slide 2
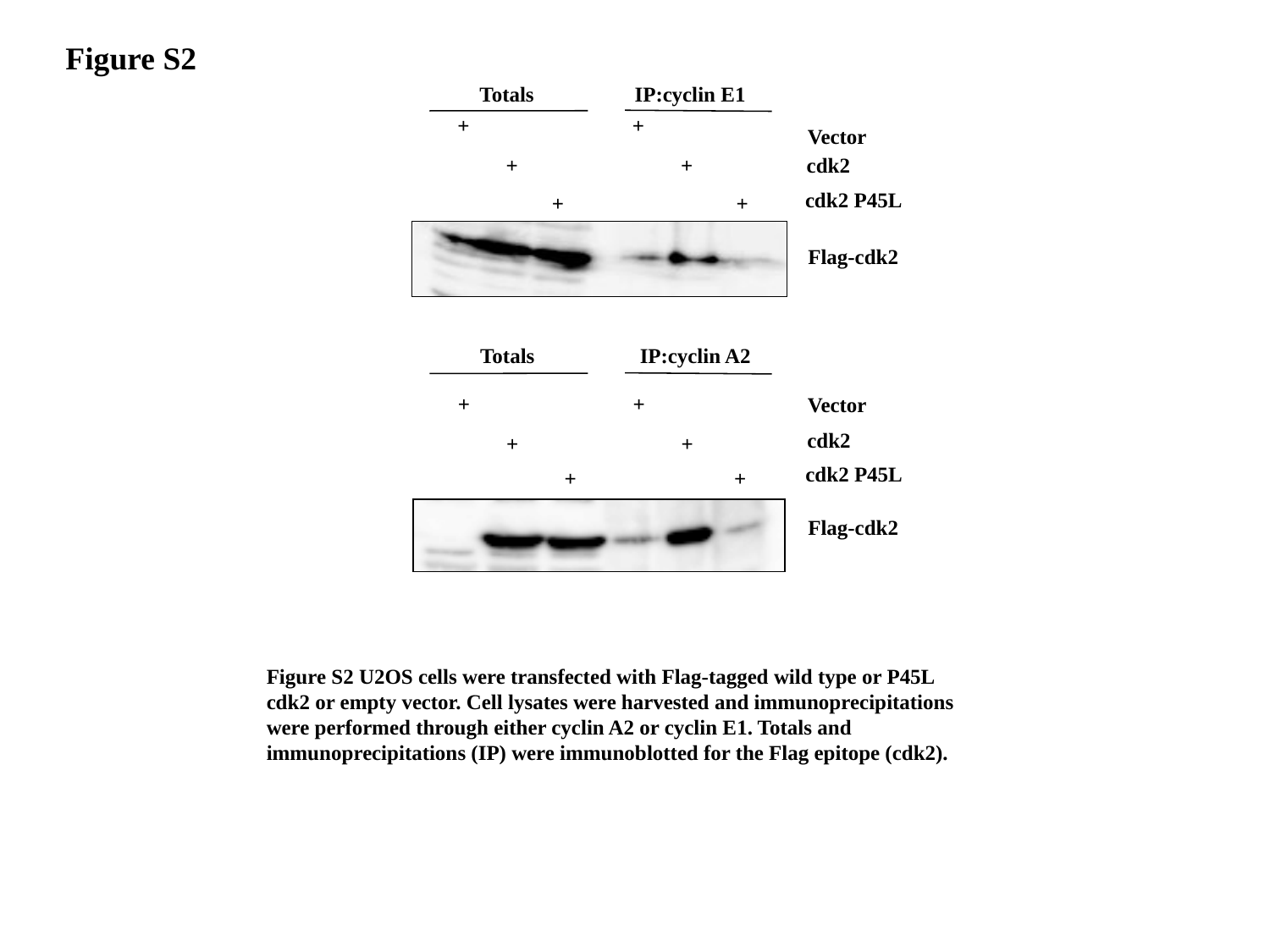

Figure S2
Totals
IP:cyclin E1
+
+
Vector
+
+
cdk2
cdk2 P45L
+
+
Flag-cdk2
Totals
IP:cyclin A2
+
+
Vector
cdk2
+
+
cdk2 P45L
+
+
Flag-cdk2
Figure S2 U2OS cells were transfected with Flag-tagged wild type or P45L cdk2 or empty vector. Cell lysates were harvested and immunoprecipitations were performed through either cyclin A2 or cyclin E1. Totals and immunoprecipitations (IP) were immunoblotted for the Flag epitope (cdk2).

## Slide 3
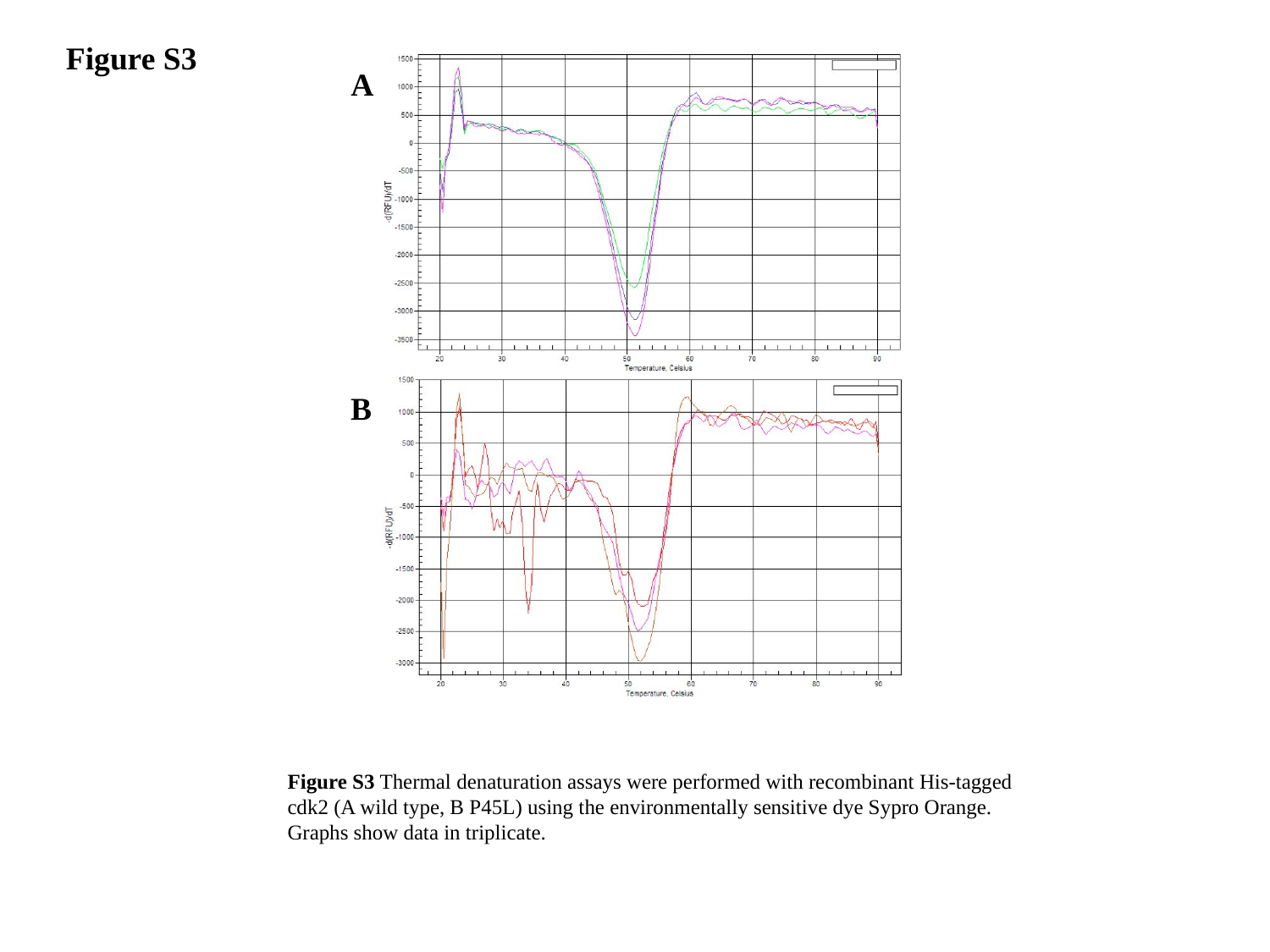

Figure S3
A
B
Figure S3 Thermal denaturation assays were performed with recombinant His-tagged cdk2 (A wild type, B P45L) using the environmentally sensitive dye Sypro Orange. Graphs show data in triplicate.

## Slide 4
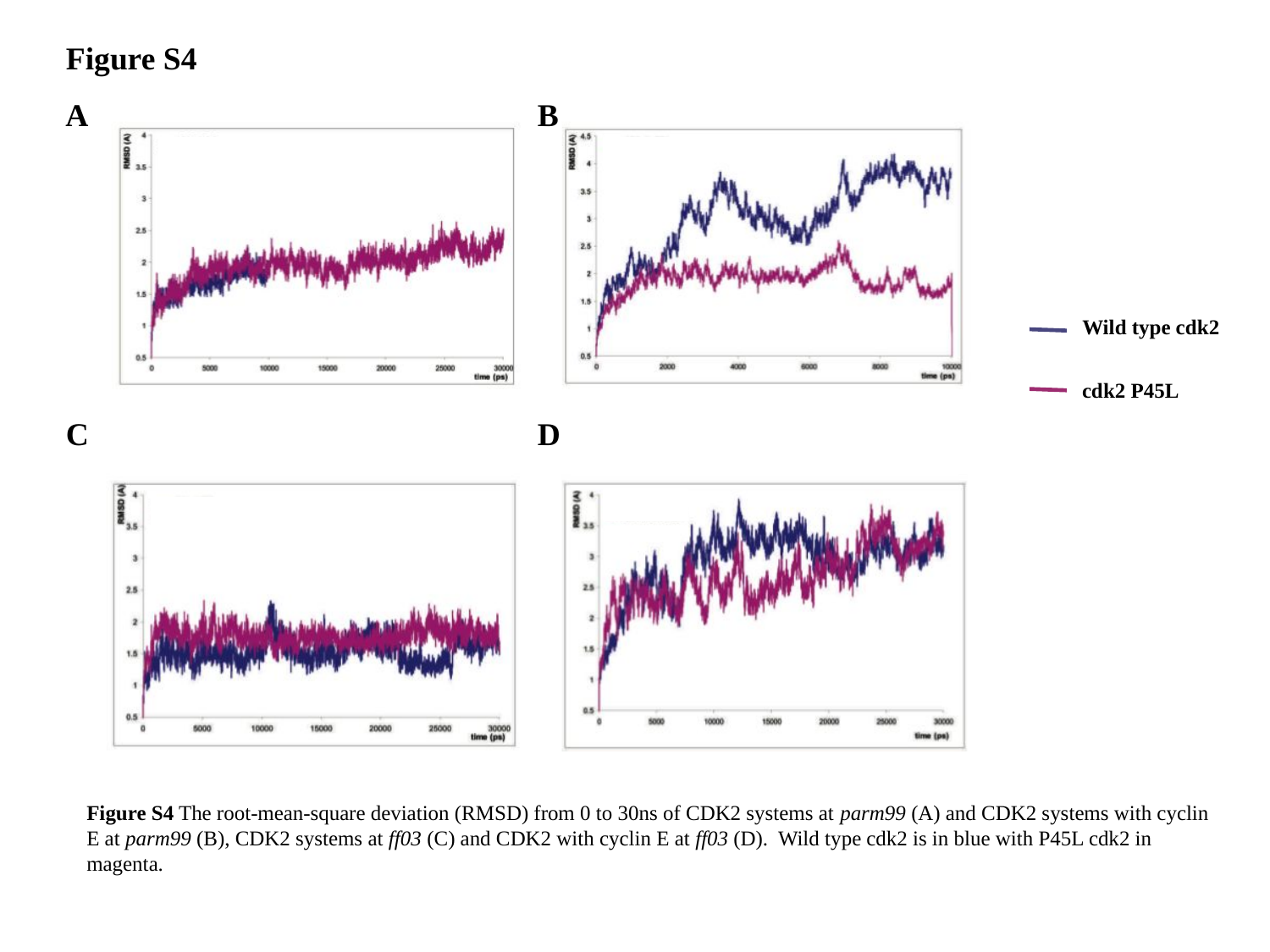

Figure S4
A
B
Wild type cdk2
cdk2 P45L
C
D
Figure S4 The root-mean-square deviation (RMSD) from 0 to 30ns of CDK2 systems at parm99 (A) and CDK2 systems with cyclin E at parm99 (B), CDK2 systems at ff03 (C) and CDK2 with cyclin E at ff03 (D). Wild type cdk2 is in blue with P45L cdk2 in magenta.

## Slide 5
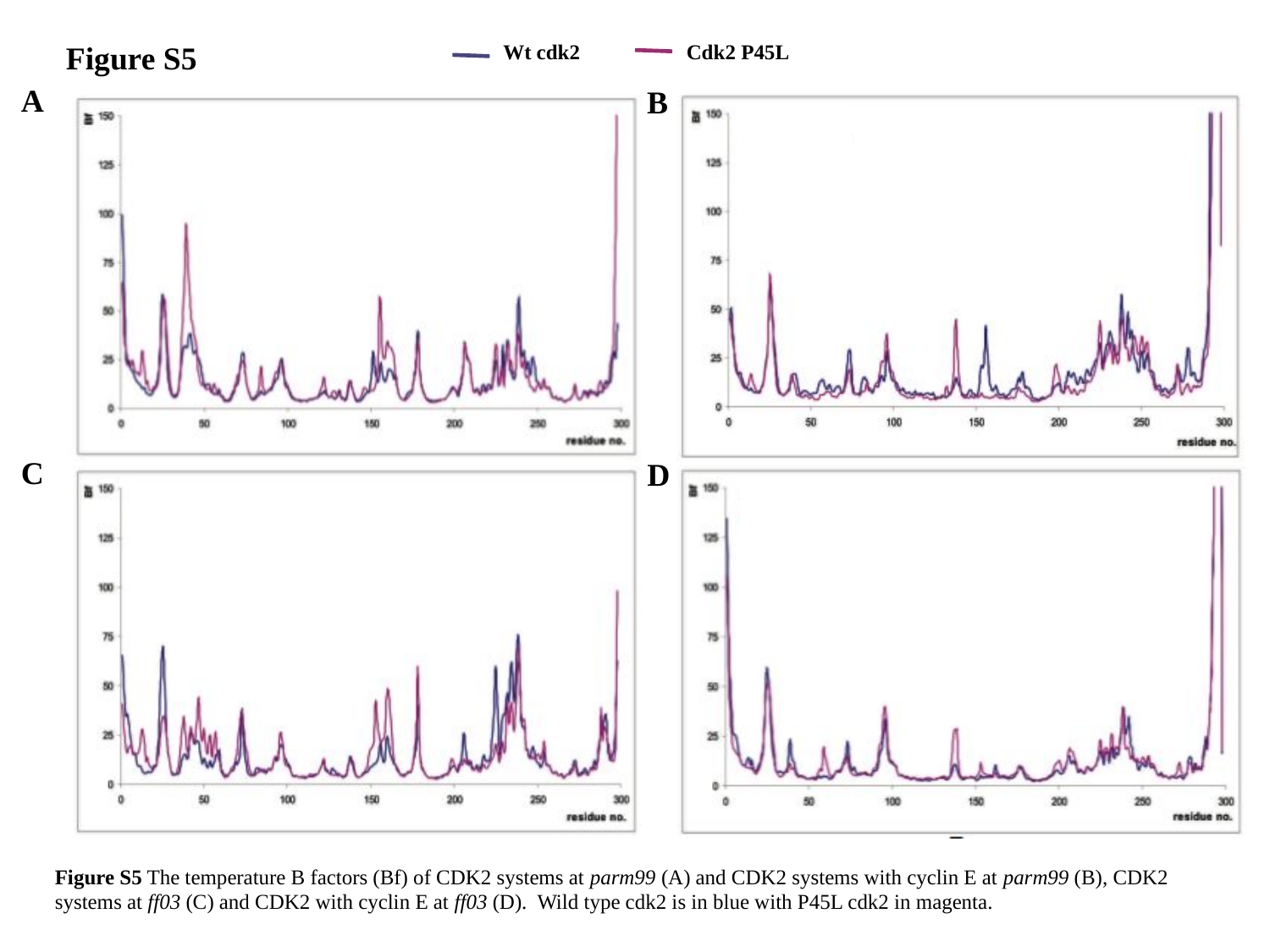

Figure S5
Cdk2 P45L
Wt cdk2
A
B
C
D
Figure S5 The temperature B factors (Bf) of CDK2 systems at parm99 (A) and CDK2 systems with cyclin E at parm99 (B), CDK2 systems at ff03 (C) and CDK2 with cyclin E at ff03 (D). Wild type cdk2 is in blue with P45L cdk2 in magenta.
